# Long-term label-free assessments of individual bacteria using three-dimensional quantitative phase imaging and hydrogel-based immobilization

**DOI:** 10.1038/s41598-022-27158-y

**Published:** 2023-01-02

**Authors:** Jeongwon Shin, Geon Kim, Jinho Park, Moosung Lee, YongKeun Park

**Affiliations:** 1 Department of Biological Sciences, Korea Advanced Institute of Science and Technology (KAIST), Daejeon, 34141 South Korea; 2 Department of Physics, Korea Advanced Institute of Science and Technology (KAIST), Daejeon, 34141 South Korea; 3 KAIST Institute for Health Science and Technology, Korea Advanced Institute of Science and Technology (KAIST), Daejeon, 34141 South Korea; 4 Tomocube Inc., Daejeon, 34051 South Korea

**Keywords:** Biological techniques, Microbiology, Optics and photonics

## Abstract

Three-dimensional (3D) quantitative phase imaging (QPI) enables long-term label-free tomographic imaging and quantitative analysis of live individual bacteria. However, the Brownian motion or motility of bacteria in a liquid medium produces motion artifacts during 3D measurements and hinders precise cell imaging and analysis. Meanwhile, existing cell immobilization methods produce noisy backgrounds and even alter cellular physiology. Here, we introduce a protocol that utilizes hydrogels for high-quality 3D QPI of live bacteria maintaining bacterial physiology. We demonstrate long-term high-resolution quantitative imaging and analysis of individual bacteria, including measuring the biophysical parameters of bacteria and responses to antibiotic treatments.

## Introduction

Long-term monitoring of bacteria is a vital task in the healthcare, bioengineering industries, and biological sciences. In clinical settings, identifying pathogenic bacteria and determining effective antibiotics against bacteria are crucial processes in treating infectious diseases^1–3^. Moreover, engineering bacteria for the efficient production of biomaterials is an active field of research^4,5^. Bacteria have also been utilized as model organisms to understand cellular mechanisms owing to their simplicity^6–8^. However, most conventional approaches to the study of bacteria have limited sensitivity because they rely on the bulk properties of bacterial colonies or high-concentration solutions. To identify pathogenic bacteria, time-consuming microbial culturing is required to secure sufficient signals in mass spectrometry-based detection devices^9^. Recently, advanced single-cell techniques have been developed and used to investigate individual bacteria^10–12^. Notably, the identification and analysis of bacteria at the single-cell level have been demonstrated in several studies^13–15^.

Quantitative phase imaging (QPI) is a label-free imaging technique that has been exploited in various biological studies^16^. QPI enables label-free, high-contrast images by measuring the optical phase delay of scattered light^16^. By enabling investigations at the single-cell level, QPI has been utilized to study blood cells^17,18^, cancer cells^19–21^, SARS-CoV-2 virus^22^, tissues^23,24^, and bacteria^13,25,26^. Due to its high-resolution, label-free, quantitative imaging capability, 2D QPI techniques have been utilized for the study of bacteria. Bacteria are studied in more detail using three-dimensional (3D) QPI, which reconstructs the 3D refractive index (RI) tomogram from multiple 2D measurements of scattered light. 3D QPI facilitates single-cell level identification of bacteria^13^, studying the response of antimicrobial photodynamic therapy^27^, quantitative tracking of polymer synthesis in bacteria^28^, and time-lapse investigation of antibiotic-treated bacteria^25^.

**Figure 1:**
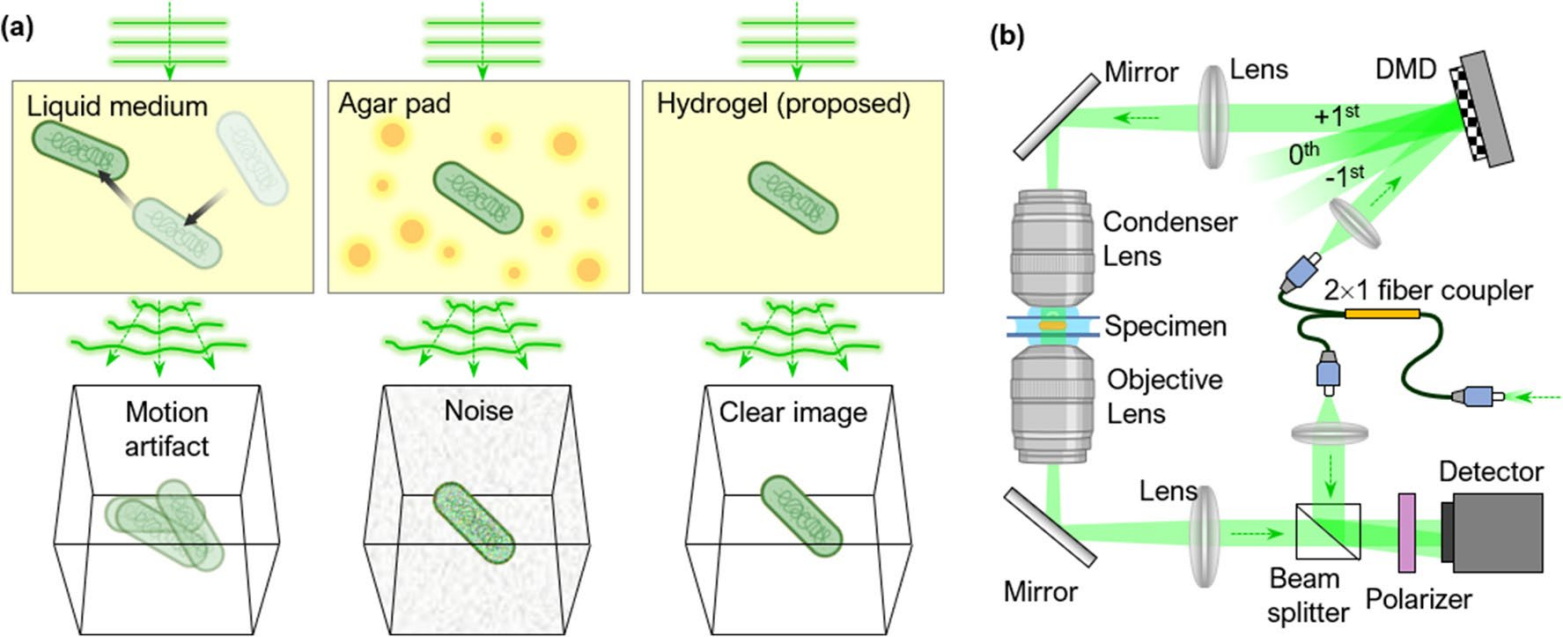
(**a**) QPI measurements of bacteria in different environments. The image quality is reduced by bacterial motility and background noise in a liquid medium and agar pad, respectively. A hydrogel-based environment prevents both issues. (**b**) Schematic of the ODT system. DMD: digital micromirror device.

However, the measurement of QPI images of individual bacteria is accompanied by experimental difficulties. One such difficulty is the Brownian motion and motility of bacteria in a liquid environment, which induce motion. The acquisition of high-quality 3D images is hindered in a liquid medium because bacterial motility generates imaging artifacts and prohibits long-term measurements [Fig. 1a]. Because 3D QPI reconstructs an RI tomogram from multiple 2D measurements, the image quality drastically drops with changes in the sample geometry during measurements. In addition, during long-term measurements, bacteria often travel from their original locations, occasionally out of the field of view (FOV).

Several studies have suggested immobilization techniques to circumvent motility-related issues when measuring individual bacteria. One technique utilizes poly-l-lysine, which is frequently used to facilitate cell adhesion to plasticware or glass surfaces^29^. However, the physiological alteration of bacteria due to poly-l-lysine restricts the utility of this technique^30^. Another bacterial immobilization technique is an agar pad^31–33^. However, when applied to 3D QPI, an agar pad produces noisy images [Fig. 1] because of the inhomogeneous distribution of RI in an agar pad.

To achieve 3D QPI measurements of bacteria with neither motion-induced problems nor background noise, we propose an experimental method that utilizes a hydrogel to improve the imaging quality. Hydrogels are cross-linked hydrophilic polymers that form optically clear extracellular matrices and are suitable materials for addressing the aforementioned issues^34,35^. Here, we experimentally demonstrate a method for imaging bacteria using hydrogel substrates and optical diffraction tomography (ODT), a 3D QPI technique^36^. Our method achieves higher image quality and stable sample immobilization compared to using a liquid medium or an agar pad environment. Moreover, our method facilitates long-term analyses of cellular features and investigation of the response to antibiotics. Based on the advantages emphasized in our study, our method provides a high-quality auxiliary technique for the study of individual bacteria.

## Results

### Measurements of high-quality ODT using a hydrogel environment

**Figure 2:**
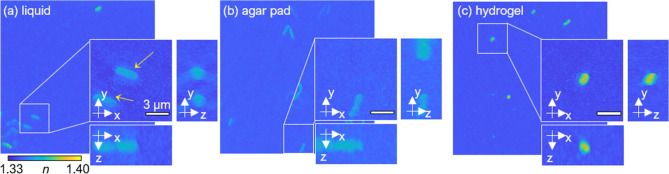
MIP visualization of 3D RI tomograms of *K. pneumoniae* in liquid (**a**), agar pad (**b**), and hydrogel (**c**).

To validate the imaging capability of the hydrogel-based environment in 3D, we compared the 3D QPI images of individual bacteria obtained in a liquid medium, agar pad, and hydrogel [Fig. 2]. We first validated that a liquid medium and hydrogel have identical background RI values, rendering the common reconstruction and analysis available for hydrogel-based measurements. We experimentally verified the homogeneous background RI distribution of liquid and hydrogel medium by comparing the phase delay maps of 2 μm SiO_2_ beads, lying in both media [Supplementary Fig. S1].

The motion artifact in the hydrogel-based environment was dramatically reduced compared with that in the liquid environment. In liquid media, large numbers of bacteria exhibited motion during measurement producing motion artifacts. The property of this motion in liquid media was further specified by analyzing the time-lapse trajectories of individual bacteria [Supplementary Fig. S2]. In this analysis, *K. pneumoniae* exhibited random motion with estimated diffusivity of 0.302 ± 0.091 μm^2^/s, while *E. coli* exhibited relatively active motion, which resonates with the known motility properties of the two species^37^. Due to the aforementioned motion, boundaries in the resulting 3D RI tomograms in a liquid medium were obscured [Fig. 2a, the yellow arrows and Supplementary Vid. 1].

The 3D RI tomograms measured under immobilizing environments exhibited a significant reduction in the motion as well as the ensuing artifact [Fig. 2b,c]. The RI tomograms of bacteria in the agar pad showed speckle-like background noise, implying inhomogeneous distribution of RIs in the agar pad [Fig. 2b and Supplementary Vid. 2]. In contrast, the hydrogel environment was able to provide clear artifact-free contrast [Fig. 2c and Supplementary Vid. 3]. To further support hydrogel’s bacteria immobilization capacity, we calculated the viscosity of a liquid medium and a hydrogel medium, which were 0.001 ± 0.000 Pa∙s and 0.286 ± 0.120 Pa∙s, respectively. Thus, our hydrogel-based protocol facilitates more accurate profiling of individual bacteria without the need for tedious data curation. For the remaining analyses in liquid media, we utilized curated tomograms without motion artifacts to address further issues, including background noise and motility.

**Figure 3:**
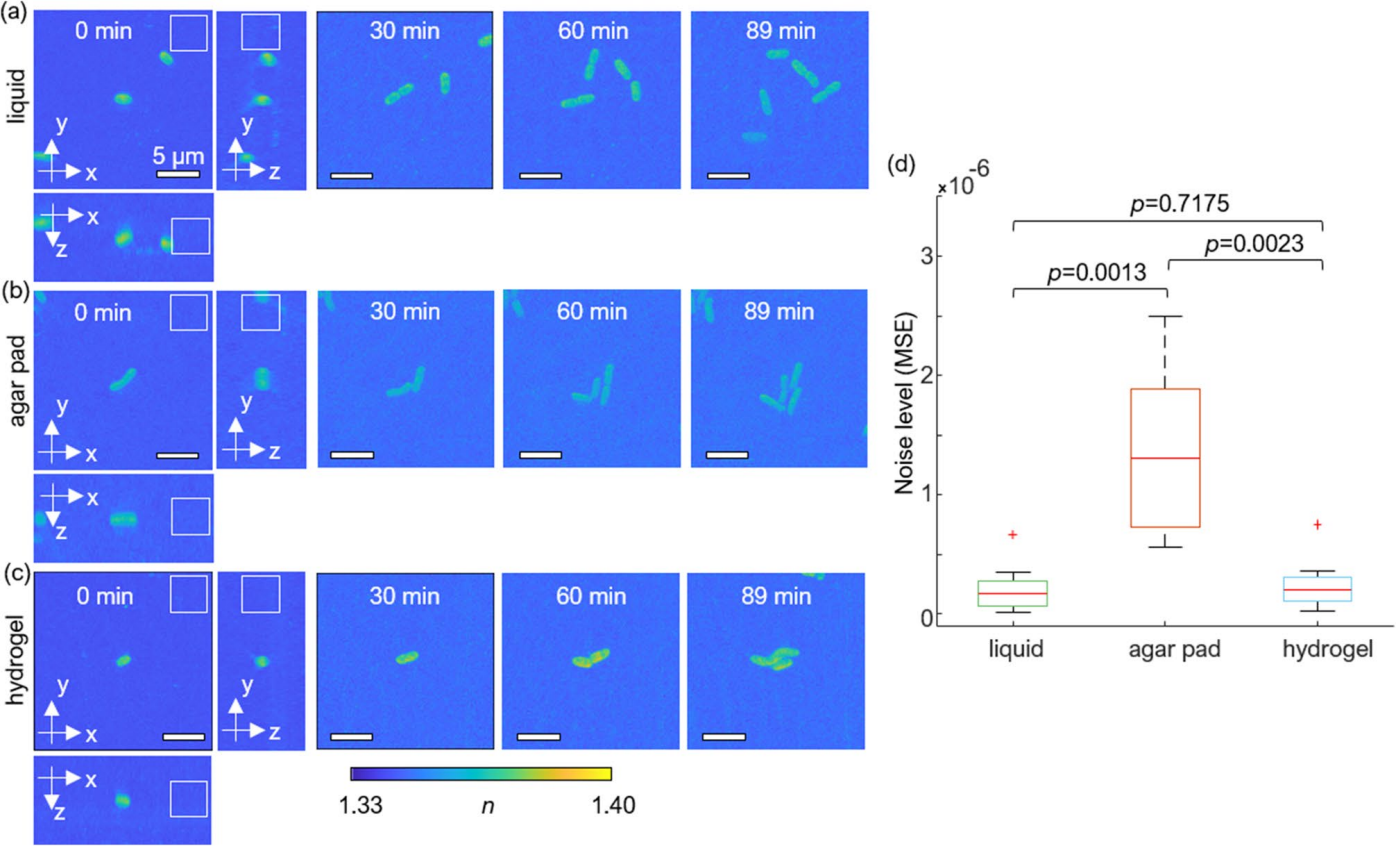
Time-lapse of MIP visualization of 3D RI tomograms of *K. pneumoniae* in different media: liquid medium (**a**), agar pad (**b**), and hydrogel (**c**). At 0 min, both 3D views of maximum RI projection and volumes marked with white squares which are used to calculate and visualize the RI distribution of background as in (**d**) are indicated. (**d**) Box plot for the noise level of background in different media; liquid medium, agar pad, and hydrogel. The background noise level was assessed from 40 × 40 × 40 pixels without samples in each tomogram. These regions are indicated with white outlines in (**a**–**c**). The mean squared errors (MSEs) of backgrounds were calculated in nine tomograms for each medium separately. The central mark indicates the median, and the bottom and top edges of the box indicate the 25th and 75th percentiles, respectively. The whiskers extend to the most extreme data points not considered outliers, and the outliers are plotted using ‘ + ’ symbol.

We examined the background noise level of the 3D QPI image and the degree of bacteria immobilization in the liquid, agar pad, and hydrogel environment [Fig. 3]. 3D RI tomograms were measured for 1.5 h in each medium environment and visualized using MIP with the corresponding bacterium centered in the image. *K. pneumoniae* in the liquid medium traveled and rotated [Fig. 3a], whereas bacteria in the agar pad and the hydrogel were fixed, enabling monitoring of growth in different directions [Fig. 3b,c]. The hydrogel environment allows for reliable visualization and measurements of bacterial growth and division. The increment of background noise over time is due to mechanical instability and medium state alteration.

Moreover, we analyzed the RI distribution of background by computing the mean squared error (MSE) [Fig. 3d]. The median values of MSE for a liquid medium, agar pad, and hydrogel were 1.69 × 10^−7^, 1.31 × 10^−6^, and 1.99 × 10^−7^, respectively. The Wilcoxon rank-sum test *p*-values were 0.0013, 0.0023, and 0.7175 for liquid and agar pad, for agar pad and hydrogel, and for liquid and hydrogel, respectively. Here, the MSE values of the background for a hydrogel system are comparable with that of the liquid medium with a high *p*-value. By comparison, the agar pad system recorded the highest MSE values of background RIs. The results show that the hydrogel is an appropriate system for bacteria immobilization while maintaining noise levels within the range of the liquid medium. This reduced noise level of the hydrogel can also lead to more precise monitoring of quantitative features including the dry mass density, compared to the agar pad. That is, due to the linear relation between the RI increment and dry mass density, variation in the quantitative features can be suppressed with precise measurement of RI.

### Biological compatibility of a hydrogel as a medium for bacteria culture

**Figure 4:**
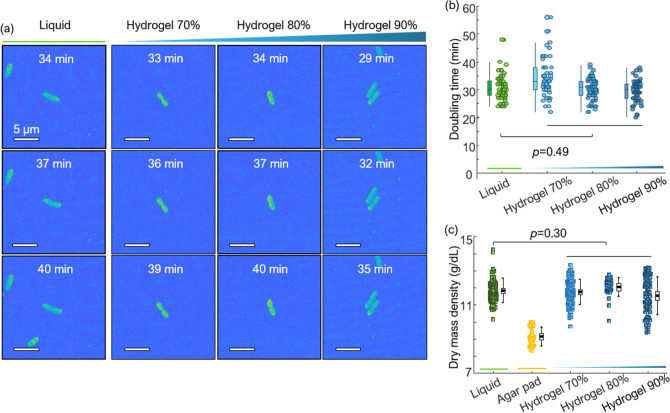
(**a**) MIP visualization of 3D RI tomograms of dividing *K. pneumoniae* over time in different media: a liquid medium, a hydrogel 70%, a hydrogel 80%, and a hydrogel 90% medium. (**b**) Distribution of *K. pneumoniae* doubling time in the different media. (**c**) Dry mass density distributions for *K. pneumoniae* at 0 min in various media. For (**b**) and (**c**) the results of the Wilcoxon rank-sum test *p*-value of the liquid and the pooled hydrogel environments are given.

We then compared bacterial division in the hydrogel environment and in the liquid medium to assess the effect of hydrogel on the physiology of bacteria [Fig. 4]. No drastic physiological changes were observed, as the doubling time of bacteria in the two environments did not vary significantly. In particular, we investigated cell morphology and the distribution of the doubling time of *K. pneumoniae* in the liquid medium and in various concentrations of the hydrogel [Fig. 4]. We acquired RI tomograms at 1 min intervals and measured bacterial growth and division in the MIP images. Timepoints at division and at 3 min before and after division were chosen for the morphological analysis [Fig. 4a]. MIP images show that bacteria retain similar morphologies, grow to a fixed size, and reproduce through binary fission without any abnormal alterations in all conditions.

In a successive step, the doubling time of *K. pneumoniae* was obtained to quantitatively determine the effects of hydrogel on biological metabolism. The division events of 57, 50, 61, and 53 bacteria were investigated for each medium environment: liquid medium, hydrogel 70%, hydrogel 80%, and hydrogel 90% medium. To improve accuracy, we performed three separate examinations for each condition.

The distributions of the doubling time of *K. pneumoniae* in the various media were comparable. The average and standard deviation of doubling time of *K. pneumoniae* in the liquid medium, hydrogel 70%, hydrogel 80%, and hydrogel 90% were 30.75 ± 4.58 min, 34.42 ± 6.95 min, 30.20 ± 3.98 min, and 29.53 ± 4.13 min, respectively [Fig. 4b]. The Wilcoxon rank-sum test *p*-value for the liquid and the pooled hydrogel media was 0.49, indicating statistical similarity. Moreover, we calculated the dry mass density distributions of the bacteria at 0 min in a liquid, an agar pad, hydrogel 70%, hydrogel 80%, and hydrogel 90% medium to further validate the constant physiological state of bacteria in hydrogels [Fig. 4c]. Total 99, 28, 91, 28, and 68 bacteria were calculated for a liquid, an agar pad, hydrogel 70%, hydrogel 80%, and hydrogel 90% medium, where the average and standard deviation values of dry mass density distributions were 11.83 ± 0.71 g/dL, 9.14 ± 0.54 g/dL, 11.75 ± 0.73 g/dL, 12.04 ± 0.56 g/dL, 11.53 ± 1.01 g/dL, respectively. The dry mass density distribution of bacteria in a liquid medium was comparable to the pooled dry mass distributions of bacteria in hydrogel 70%, hydrogel 80%, and hydrogel 90% medium with the Wilcoxon rank-sum test *p*-value of 0.3 [Fig. 4c]. In contrast, the dry mass density distribution of bacteria in an agar pad represents a decrement compared to the others, with the Wilcoxon rank-sum test *p*-value of 8.30 × 10^−36^ between a liquid medium environment. We also note that the precision of the dry mass measurement is directly related to the experimental precision of RI and the imaging resolution, which are decisive for the dry mass density and the volume, respectively.

### Cell tracking efficiency of hydrogel when yielding cellular features

**Figure 5:**
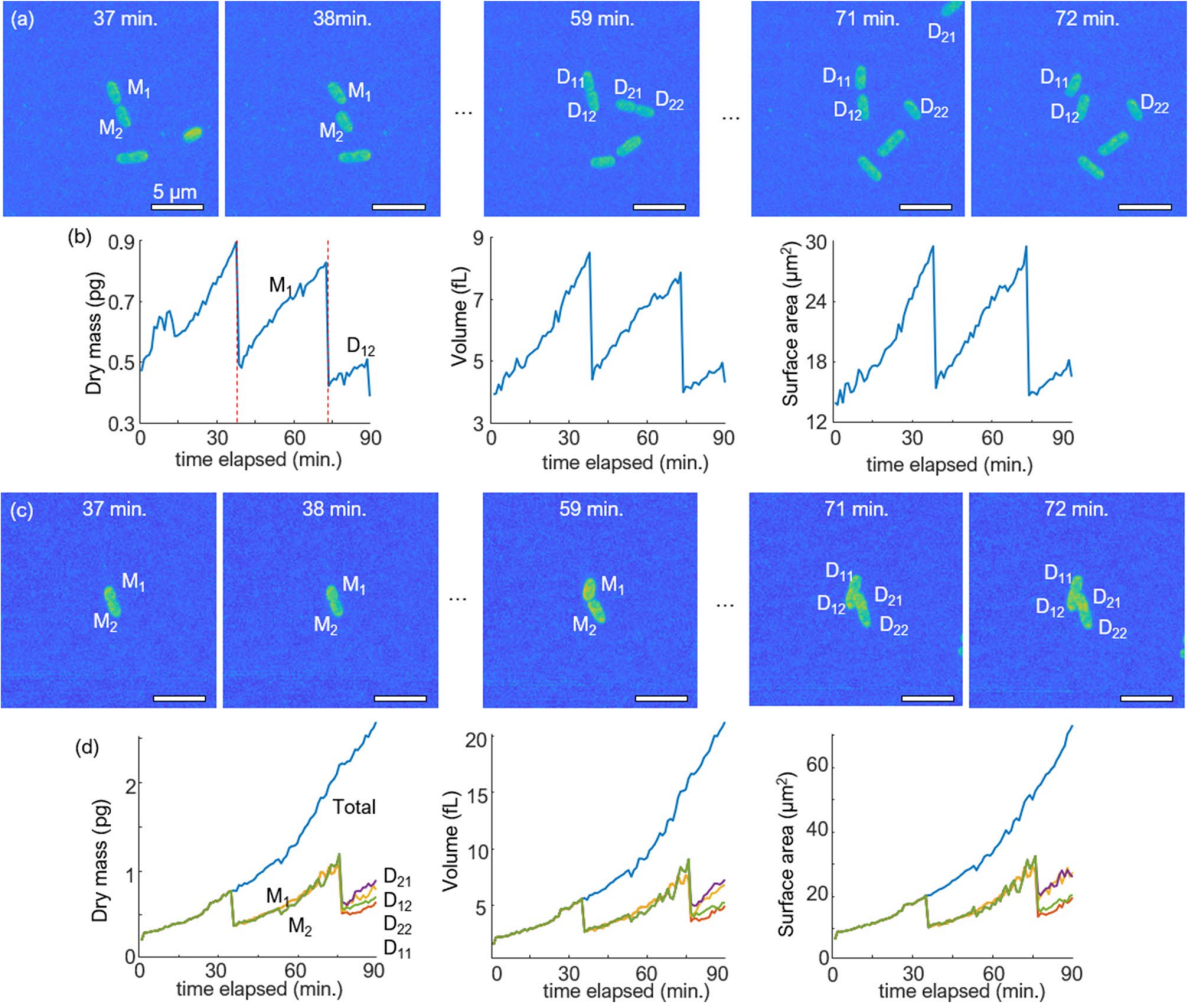
MIP visualization and quantitative analysis of 3D RI tomograms of *K. pneumoniae* in the liquid (**a**,**b**) and hydrogel (**c**,**d**) medium. The quantitative analysis includes cellular dry mass, volume, and surface area extracted from each snapshot tomogram. (**a**,**c**) M_1_ and M_2_ refer to the mother cells while D_11_, D_12_ and D_21_, D_22_ refer to two daughter cells from each mother cell. In (**a**), one daughter cell (D_21_) can be seen at the upper right corner traveling out of the field of view at 71 min. (**b**) Time points showing sudden drops in cellular dry mass of *K. pneumoniae* are marked with red dashed lines. In (**d**), pooled data of all daughter cells and single-cell analyses are presented.

Our hydrogel-based protocol facilitated the 1.5 h monitoring of a single bacterium, allowing the investigation of morphological and biochemical features over time. The following cellular features were retrieved and analyzed using 3D RI tomograms: cellular dry mass, volume, and surface area. The temporal changes in these features were plotted to quantitatively assess bacterial growth in both the liquid and hydrogel media [Fig. 5b,d].

The growth of *K. pneumoniae* could be continuously tracked in the 80% hydrogel medium; this was difficult to accomplish in the liquid medium because of bacterial motility. The values of the cellular features in the liquid medium show sudden drops at certain points [Fig. 5b]. A review of acquired images showed two cases that hindered continuous growth measurements in the liquid medium. In the first case, the motility and rotation of the daughter cells rendered it difficult to trace their origins. In the second case, one of the daughter cells moved out of the field of view [Fig. 5a, D_21_ at 71 min]. In contrast, the hydrogel medium becomes a practical environment for continuously tracking and analyzing cellular features. Bacteria are trackable and well immobilized for all daughter cells [Fig. 5c]. Thus, the pooled cellular features of the daughter cells in the hydrogel medium displayed a constant increase without sudden drops. In addition, analysis of the features of each daughter cell in the hydrogel was feasible [Fig. 5d]. The cellular features of single daughter cells clearly show two consecutive cell divisions, while analysis of the features of the cell clusters can potentially facilitate diagnosis.

### Further application for imaging-based antimicrobial susceptibility testing

**Figure 6:**
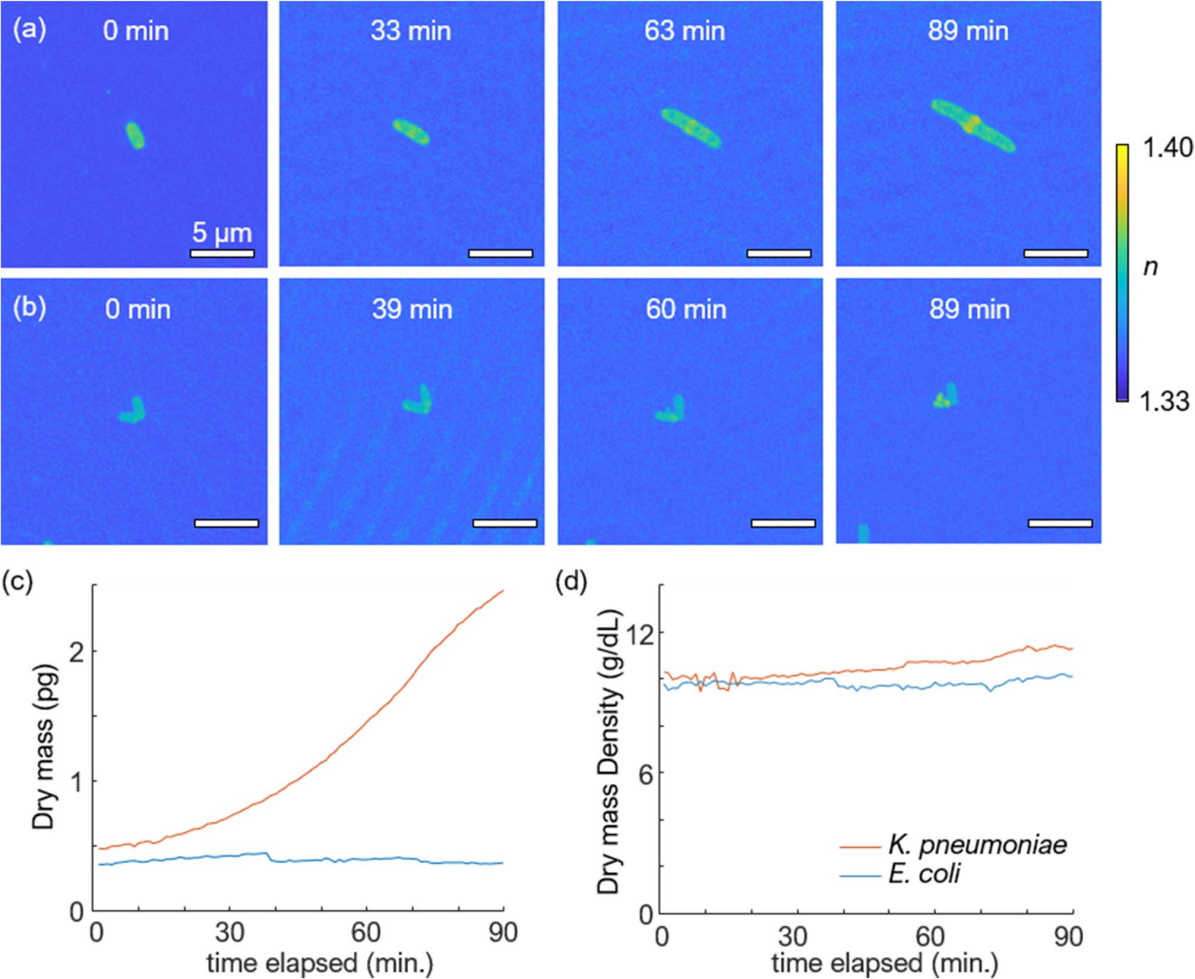
(**a**) MIP visualization of 3D RI tomograms of *K. pneumoniae* (**a**) and *E. coli* (**b**) treated with 200 μg/mL of ampicillin. Quantitative analysis of dry mass (**c**) and dry mass density (**d**) over time for *K. pneumoniae* and *E. coli* were plotted*.*

Finally, we demonstrated the potential application of hydrogel as an imaging medium for imaging-based antimicrobial susceptibility testing (AST)^38,39^ [Fig. 6]. We treated *K. pneumoniae* and *E. coli* with 200 μg/mL of ampicillin in the 80% hydrogel medium and measured 3D RI tomograms for 90 min to assess whether the hydrogel-based environment is suitable for evaluating bactericidal responses^25^.

As a result of ampicillin treatment, alterations of *K. pneumoniae* in the biochemical and morphological properties were identified. First, the treatment of ampicillin led to an immediate drop in the dry mass density. The dry mass density in an 80% hydrogel medium treated with ampicillin at 0 min was significantly decreased to 10.33 g/dL [Fig. 6c] while that in an 80% hydrogel medium without ampicillin had an average value of 12.04 ± 0.56 g/dL [Fig. 4c]. Also, a prolonged elongation of *K. pneumoniae* was observed. Even after the duration of time that is sufficient for cell division, the cell length kept increasing without division as shown in the MIP images [Fig. 6a, 63 min]. The formation of a high RI bulge in the middle of the cell was also visible [Fig. 6a, 89 min], which has been reported in previous studies as the result of the inhibited transpeptidase^40,41^.

Figure 6b shows the MIP of a single *E. coli* treated with ampicillin. Over time, a bulge formation can be detected along with no increment of dry mass values [Fig. 6b, 60 min and Fig. 6c]^40^. Based on these observations, we conclude that the hydrogel can be used successfully to image the physical responses of bacteria to antibiotics.

## Discussion and summary

We propose and demonstrate a protocol for 3D QPI of bacteria that does not suffer from motion-induced artifacts or background noise. Our protocol utilizes hydrogel as part of the loading medium and benefits from the formation of optically clear extracellular matrices^34,35^. The hydrogel facilitates high-quality 3D QPI measurements of bacteria by immobilizing bacteria without generating noise. Moreover, the physiological characteristics of samples in a hydrogel system are invariable and readily accessible for analysis as indicated by our statistical analysis of the doubling time and dry mass density. The biocompatible and steady environment created by our protocol enabled long-term quantitative measurements. Further experiments also suggest the feasibility of our protocol in practical applications, such as measuring the efficacy of antibiotics in a hydrogel environment based on 3D QPI. We believe that our protocol would facilitate the applications in which 3D morphology measurement is crucial as illustrated in the following.

Identification of pathogenic bacteria is a potential application of the proposed method. Time-consuming microbial culture is an obstacle to early appropriate antibiotic treatment due to the need for ensemble behavioral data of myriad bacteria upon antibiotics^42^. Single-cell techniques, including QPI, aid in overcoming this limitation by measuring responses from individual bacteria^13,43^. And our method can support bacterial identification with QPI by providing a more stable environment for measurements. Our method can be further applied in a high-quality imaging pipeline for the QPI-based identification of bacteria samples. An earlier study has demonstrated the rapid identification of bacteria using 3D QPI and deep learning^13^, and we believe that our protocol can boost data acquirement and enhance the performance of such techniques by reducing motion artifacts and enabling long-term imaging. In addition, the classification of bacteria based on biomarkers will also be viable using our method^43^, as it provides a more stable acquisition of biomarkers, including dry mass and volume. Therefore, our protocol will propel 3D QPI for rapid bacterial identification.

The study of bacterial responses in different environments is another field to which our protocol can contribute. One practical application of this method is AST. AST is used to assess the antibiotic resistance of specific bacteria. This information is crucial for medical doctors to determine the types of antibiotics used for infection treatment^44^. Traditional AST utilizes populational information of bacteria when measuring their susceptibility to antibiotics^45^. Our protocol can be implemented for not only population-based AST^24^, but also for assessing the drug susceptibility of individual bacterium, which has been indicated as a rapid alternative to the conventional time-consuming approaches^28^. A commercialized label-free individual bacteria AST method evaluates samples in an agarose-based environment using bright-field microscopy. This method has the advantage of label-free imaging but still entails a day-long delay owing to the requirement of incubation^46,47^. This long turnaround time is not present in 3D QPI-based AST due to the high sensitivity and accessibility to quantitative biophysical properties. In visualizing the morphology, 3D QPI provides further contrast and sensitivity than bright-field microscopy, as cells typically induce more diffraction than absorption in the visible wavelength. Furthermore, 3D QPI provides not only the morphological information as bright-field microscopy does, but also additional quantitative information, including RI distribution, dry mass, and dry mass density, which are suitable for single-cell analysis such as cell death^48^. Using our approach, 3D QPI monitoring of individual bacteria can reveal antibiotic susceptibility within approximately an hour. The application of our method in 3D QPI is especially efficient when it comes to AST for unculturable or hardly culturable bacteria. For instance, *Treponema pallidum* subsp. *pallidum* and *Synergistetes* strains, which are the major causative agents of syphilis and periodontal diseases^49,50^, are arduous to culture in the laboratories as they require long and unique culture conditions, special reagents, and co-culturing with specific cell lines. Those bacteria can be tested in time- and resource-efficient ways by accurately analyzing individual bacteria with 3D QPI and our protocol. Therefore, we envision that our protocol can accelerate label-free AST by eliminating the need for time-consuming cultivation procedures. Furthermore, a more efficient AST based on 3D QPI and our method can be realized by introducing microfluidic techniques. By forming drug gradients in a microfluidic chip, an on-chip AST based on 3D QPI monitoring can be facilitated.

Our method can also benefit studies of microbial biopolymer synthesis by quantitatively monitoring the production levels of each cell. Bacteria can be used as cell factories for biopolymers, which are then used in medical and industrial applications^4^. By monitoring individual biopolymer production levels non-invasively, more efficient conditions for biopolymer production can be applied at medical and industrial levels^28^.

Moreover, our protocol can aid in cellular mechanism studies of individual bacteria. Single-cell analysis with 3D QPI based on our protocol would enable the phenotyping of individual bacterium depending on their cellular mechanism states. Seemingly uniform bacterial populations for research may differ in cell cycle states, and genotypic and phenotypic subpopulations to their neighbors^51^. Our method can facilitate investigating heterogeneous cellular physiology at single-bacterium resolution by providing stable long-term analysis for both qualitative morphology and quantitative RI information of individual live cells^16,52^.

We plan to investigate the capability of our method extensively. Although we measured each specimen within 1.5 h, with more precise maintenance of temperature and humidity, a much longer investigation would be possible. In addition, a hydrogel-based environment can be implemented in a shorter time using different polymer compositions, as already suggested by several studies^53,54^. To extensively utilize the proposed method for AST, the influence of hydrogel in the diffusion of antibiotics should be investigated^55^. Ampicillin, which was utilized in our demonstration, is unlikely to be impeded during diffusion due to its small molecular weight. However, recent studies have also suggested macromolecular antibiotics, of which the diffusion dynamics may vary due to mesh environments such as hydrogel^56^.

## Methods

### Sample preparation

The bacterial strains *Klebsiella pneumoniae* (clinical isolates from the Asian Bacterial Bank of the Asia Pacific Foundation for Infectious Diseases) and *Escherichia coli (*ATCC25922) were stored in a − 70 °C deep freezer. Five microliters of the bacterial sample were added to 1000 μL of tryptic soy broth (TSB; MBcell) and incubated for 1 h at 37 °C in a 5% CO_2_ shaking incubator. The stabilized mixture (100 µL) was spread on tryptic soy agar (TSA) plates and incubated for 24 h at 37 °C in a 5% CO_2_ incubator. The TSA plates were stored at 4 °C and used within a week. Before the experiments, a colony was selected, resuspended in 1000 μL TSB, and incubated for 24 h at 37 °C in a 5% CO_2_ shaking incubator.

### Hydrogel preparation

Hydrogels were formed by the combination of HyStem and Extralink (HYS020, Sigma-Aldrich, Saint Louis, Missouri, USA) with a volume ratio of 4:1. HyStem and Extralink are thiol-modified hyaluronan and thiol-reactive crosslinkers, respectively. HyStem and bacterial cultures were incubated in a 37 °C water bath before use. For the 70% hydrogel sample, we mixed 14 μL of HyStem, 7.5 μL of bacteria culture, and 3.5 μL of Extralink in one Eppendorf (EP) tube and 14 μL of HyStem, 7.5 μL of TSB, and 3.5 μL of Extralink in another EP tube. For the 80% hydrogel sample, we mixed 16 μL of HyStem, 5 μL of bacterial culture, and 4 μL of Extralink in one EP tube and 16 μL of HyStem, 5 μL of TSB, and 4 μL of Extralink in another EP tube. For the 90% hydrogel sample, we mixed 18 μL of HyStem, 2.5 μL of bacterial culture, and 4.5 μL of Extralink in one EP tube and 18 μL of HyStem, 2.5 μL of TSB, and 4.5 μL of Extralink in another EP tube. We then placed a 20 mm × 20 mm cover glass on a TomoDish (Tomocube Inc., Daejeon, Korea), gently pressed only the sides of the glass to secure it, and incubated the chamber on a 37 °C hot plate. Ten microliters from each EP tube were added to each side groove of the TomoDish. Finally, the chamber was sealed by adding 10 μL of mineral oil to the grooves. TomoDish was incubated for 10 min on a hot plate at 37 °C to fully solidify the hydrogel. Approximately 20 min were required for the sample preparation in a hydrogel medium, including incubation for the solidification of a hydrogel. HyStem and Extralink were stored at − 20 °C when not in use.

### Agar pad preparation

Agar pads are agarose gels that solidify in thin cuboid forms. Here, we adopted the gold standard protocol for bacteria imaging on an agar pad, in which bacteria are settled between an agar pad on the bottom and a glass cover glass on top^57–59^. We prepared a 20 mm × 20 mm parafilm and excised a square hole of 12 mm × 12 mm. The parafilm was placed on a TomoDish and fixed by partial melting on a hot plate at 70 °C. Fully melted 1.5% agarose gel (214010, BD, Franklin Lakes, New Jersey, USA) was poured onto the TomoDish and coverslipped. After the agarose gel was fully solidified, the coverslip was removed without generating any tears or wrinkles on the agar pad. Next, 5 μL of a sample was dropped on the agar pad, which was then gently covered with a new coverslip. Finally, the chamber was sealed by adding 10 μL of mineral oil to the grooves and incubated for 10 min on a 37 °C hot plate. Approximately 30 min were required for the sample preparation on an agar pad including the incubation.

### RI diffraction tomography

To acquire a 3D RI tomogram of the bacteria, we used an ODT instrument (HT-2H, Tomocube Inc., Daejeon, Korea) with a live cell imaging chamber (Tomochamber, Tomocube Inc., Daejeon, South Korea) which provides a moist environment and maintains the temperature and CO_2_ concentration of the sample to 37 °C and 5%, respectively. The variability in the RI of water can be expressed with an empirical equation along with temperature change and empirical coefficients but is negligible in our measurement condition^60,61^. This optical system is based on a Mach–Zehnder interferometric microscope with a digital micromirror device (DMD) to control the angle of the sample beam [Fig. 1b]^62^. A coherent laser beam (*λ* = 532 nm in vacuum) was split into sample and reference beams using a 2 × 2 single-mode fiber optic coupler. The DMD controls the angle of the sample beam impinging on a sample. The imaging exposure time is around 0.15 s considering 49 DMD patterns and 3.019 ms of camera shutter time length. The beam diffracted by a sample is imaged on the camera plane, where it interferes with the reference beam. Using the phase-retrieval algorithm, the phase and amplitude images were retrieved from each interferogram. The 3D RI distribution was reconstructed from various 2D holograms of the sample from different illumination angles and by inversely solving the Helmholtz equation with the Rytov approximation. The theoretically calculated lateral and axial spatial resolutions of the optical imaging system were 110 nm and 360 nm, respectively^63^. Because of the limited numerical aperture of condensers and objective lenses, side-scattering signals were not collected, leading to image quality degradation of the reconstructed 3D RI tomograms. To resolve this missing cone problem, an iterative regularization algorithm based on a non-negative constraint was used^64^. The detailed implementation and reconstruction code were as published previously^65,66^.

## Supplementary Information

Supplementary Video 1.

Supplementary Video 2.

Supplementary Video 3.

Supplementary Information 1.

## Data Availability

The datasets used and/or analysed during the current study available from the corresponding author on reasonable request.
