# Supplementary material for: Long-term label-free assessments of individual bacteria using three-dimensional quantitative phase imaging and hydrogel-based immobilization
Source: Sci Rep. 2023 Jan 2;13:46. doi: 10.1038/s41598-022-27158-y (PMC9806822; doi:10.1038/s41598-022-27158-y)
Supplement: Supplementary file 4 — Supplementary Information 1. [file 41598_2022_27158_MOESM4_ESM.docx]

**Supplementary Information**

**Long-term label-free assessments of individual bacteria using three-dimensional quantitative phase imaging and hydrogel-based immobilization**

Jeongwon Shin^1†^, Geon Kim,^2,3†^ Jinho Park^2^, Moosung Lee^2,3^, and YongKeun Park^2,3,4*^

^1^Department of Biological Sciences, Korea Advanced Institute of Science and Technology (KAIST), Daejeon, 34141, South Korea

^2^Department of Physics, KAIST, Daejeon, 34141, South Korea

^3^KAIST Institute for Health Science and Technology, KAIST, Daejeon 34141, South Korea

^4^Tomocube Inc., Daejeon, 34051, South Korea

^†^ These authors contributed equally to this work.

Corresponding author: YongKeun Park

[yk.park@kaist.ac.kr](mailto:limsoo@snu.ac.kr)


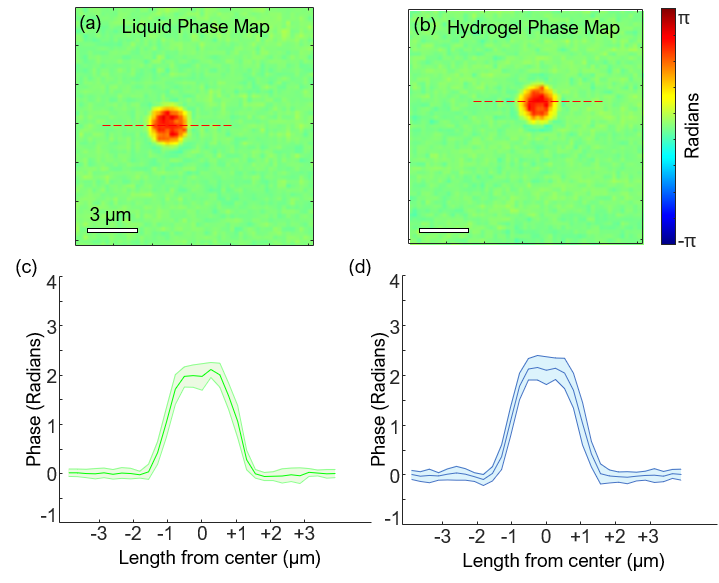


Fig. S1. Hydrogel background RI value comparison. Phase maps with 2 μm SiO_2_ beads were measured for both a liquid medium (a) and a hydrogel medium (b). We calculated the phase distribution at a distance of ± 4 μm from each bead center, as indicated by red dotted lines. Total 15 and 24 beads were measured and plotted respectively from a liquid (c), and a hydrogel medium (d). Solid lines are the average values and shadow areas represent the standard deviation.


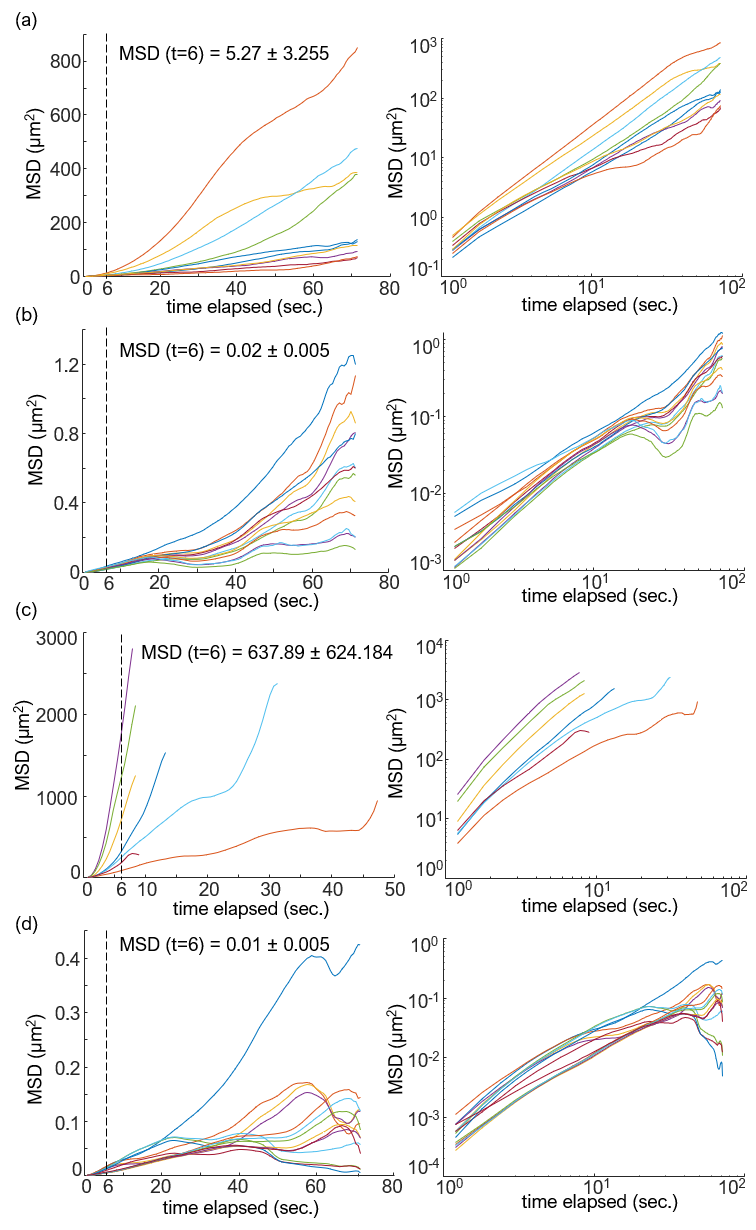


Fig. S2. Plots for mean squared displacement (MSD) over time and their log-log plots. Plots for *K. pneumoniae* in a liquid (a) and a hydrogel 80% (b) environment and for *E. coli* in a liquid (c) and a hydrogel 80% (d) environment were presented. For every MSD over time plots, the average and standard deviation values for the pooled bacteria at 6 seconds were indicated.

**Captions for Supplementary Videos**

**Supplementary Vid. 1**

The 3D view of bacteria in a liquid medium. Scale bar, 5 μm

**Supplementary Vid. 2**

The 3D view of bacteria in an agar pad. Scale bar, 5 μm

**Supplementary Vid. 3**

The 3D view of bacteria in a hydrogel medium. Scale bar, 5 μm

**Hydrogel background RI measurement**

We measured the background RI of a hydrogel in comparison with a liquid medium using 2 μm SiO_2_ beads (81108-5ML-F, Sigma-Aldrich, Saint Louis, Missouri, USA). We made 0.1% bead solution by mixing beads with deionized water. For a liquid medium bead sample, we applied 10 μL of TSB and 10 μL of bead solutions to each side groove of the TomoDish covered with 20 mm × 20 mm cover glass. For a hydrogel medium bead sample, we mixed 16 μL of HyStem, 5 μL of bead solutions, and 4 μL of Extralink in one EP tube and 16 μL of HyStem, 5 μL of TSB, and 4 μL of Extralink in another EP tube. Then we added 10 μL from each EP tube to each side groove of the TomoDish covered with 20 mm × 20 mm cover glass. TomoDish was incubated for 10 min on a hot plate at 37°C to fully solidify the hydrogel. After measuring individual beads with HT-2H, we retrieved the phase maps with the incident light entering vertically and measured the phase distributions to ± 4 μm around the center of each bead [Supplementary Fig. S1(a) and S1(b)]. 15 and 24 beads were measured and visualized for a liquid medium and hydrogel, respectively.

**Cell doubling time analysis**

Cell doubling time analysis was performed through manual inspection of the time-lapse maximum intensity projection (MIP) images. MIP is a projection method for 3D data that visualizes only the maximum RI values among RI from every parallel 3D data plane to the visualization plane. In each tomogram, the individual bacteria were automatically segmented using a threshold-based algorithm. The time point for each cell division was marked by manually inspecting the visualization of the segmentation masks. To secure highly precise statistical comparisons, for each bacterium we standardized the starting time point of the analysis. Specifically, the analysis started immediately after the first division was observed. In this way, variance in the initial cell phase between individual bacteria was excluded. We defined the timepoint of cell division as the time point when an additional segment of the cytoplasm occurred for the first time by division. This definition excludes the error of misrecognizing the time point when two daughter cells overlap and separate again in the MIP image. To avoid misrecognizing the division point even if two daughter cells happened to overlap again, we used the interval between two adjacent cell division time points as the doubling time. For accuracy, measurements were performed three times independently. The analysis of cells in the liquid medium involved relatively immobilized cells.

**Cellular features analysis**

To measure cellular features, voxels with RI values higher than a set RI value of 1.350 were selected by masking the sample area. The dry mass of each cell was calculated using the linear relation between RI and cellular dry mass^1,2^: *n*(***r***) − *n*0 = *α* × *C*(***r***). Here *n, n0, α,*and*C* refer to the RI of a sample, the RI of the background medium, RI increment (RII), and protein concentration respectively. RII, which is an increment of an RI in a solution per an increment of concentration in a solute, determines the concentration of each point. The typical RII value for proteins is known to be 0.185 mL/g ^3^. Since many bacteria have proteinaceous organelles ^4^, we utilized this typical RII value for proteins in our analysis. The cellular dry mass values were calculated by the total sum of the concentration of each voxel multiplied by its volume ^4^. The cellular volume was estimated by the number of pixels and image resolution. Other physiological features were obtained in a similar manner. For the analyses of cells in the liquid medium, measurements were performed on the relatively immobilized cells.

**Kinetic analysis of bacterial motion**

To quantitatively address the motion of bacteria and immobilization by hydrogel, we calculated mean squared displacement (MSD) over time in a liquid and a hydrogel medium. The MSD was dramatically reduced in the hydrogel medium compared to the liquid medium. For *K. pneumoniae,* which are non-motile bacteria, the averaged MSD at 6 seconds was decreased by 263 times in a hydrogel medium, compared to a liquid medium [Fig. S2 (a) and(b)]. In the case of *E. coli,* which are motile bacteria, the averaged MSD at 6 seconds decreased by 63,789 times in a hydrogel medium, compared to a liquid medium [Fig. S2 (c) and (d)]. MSDs of *E. coli* are significantly reduced in a hydrogel medium as they are capable of flagellar locomotion in a liquid medium.

We also characterized the type of bacterial motion in a liquid medium, by investigating the logarithmic relation between MSD and time. It is known that bacteria that undergo only the Brownian motion manifest linear relation between MSD and time, while those with additional driving force go through the active Brownian motion where the MSD is governed by a higher-order power law relation to time ^5^. The slopes of the linear fits in the logarithmic plots of MSD over time were evaluated to estimate the power law relation between MSD and time. The average power for *K. pneumoniae* and *E. coli* were 1.23 ± 0.16 and 1.74 ± 0.22, respectively [Fig. S2(a) and (c)]. Therefore, the dynamic nature of *K. pneumoniae* was in high proximity to the Brownian motion, whereas *E. coli* displayed the active Brownian motion. The diffusivity of *K. pneumoniae* in three dimensions, under the assumption of the Brownian motion, was decreased from 0.302 ± 0.091 μm^2^/s to 0.002 ± 0.001 μm^2^/s in a hydrogel compared to a liquid medium. It is equivalent to the increment of the viscosity from 0.001 ± 0.000 Pa∙s to 0.286 ± 0.120 Pa∙s from a liquid to hydrogel environment, which are calculated by assuming prolate ellipsoidal morphology of *K. pneumoniae*. These values of viscosity estimated from the bacterial motions are within the range of theologically measured values from the previous studies ^6,7^.

**Methods for kinetic analysis**

To measure mean squared displacement (MSD) over time for both motile and non-motile bacteria in a liquid and a hydrogel medium, we measured *K. pneumoniae* and *E. coli* in a liquid and 80% hydrogel medium separately. The time interval, total duration of imaging, and field-of-view (FOV) were 0.6 sec, 72 sec, and 60 × 60 μm^2^, respectively. A total of 10, 13, 7, and 14 bacteria were analyzed for *K. pneumoniae* in a liquid medium, *K. pneumoniae* in a hydrogel medium, *E. coli* in a liquid medium, and *E. coli* in a hydrogel medium case, respectively.

The trajectory of each bacterium was tracked by manually pursuing the centroid position. After reconstructing the tomograms of the samples, voxels with RI values higher than a set RI value of 1.345 were selected by masking the sample area. Out of the masked sample area, we manually segmented the 3D mask of each bacterium and calculated the centroid at every frame.

MSD for a time interval $\tau$ is defined as the average of the squared displacement of the position during the given time interval.

$$MSD(\tau)\equiv\left\langle\left|\vec{r}(t)-\vec{r}(t-\tau)\right|^{2}\right\rangle_{t}$$

where $\vec{r}$refers to the position. In the case of Brownian motion in a single dimension, MSD exhibits a linear relation with the time interval $\tau$,

$$MSD(\tau)=2D\tau$$

where $D$ is the diffusivity. The diffusivity, assuming the Brownian motion of *K. pneumoniae*, was obtained according to this relation, using the y-intercepts of the logarithmic plots for MSD and time. Furthermore, the viscosity was estimated from the diffusivity and size of *K. pneumoniae*, by approximating each bacterium as a prolate ellipsoid. Assuming a prolate spheroid object, the diffusivity along an axis is expressed proportionally to the inverse of the drag coefficient $\gamma$

$$D=\frac{k_{B}T}{\gamma}$$

where $k_{B}$ and $T$ denote the Boltzmann constant and temperature ^8,9^. In a fluid with a low Reynolds number, the translational drag coefficient of a spheroid can be written as

$$\gamma=6\pi\eta bG$$

in which $G$ is the geometric factor that accounts for the difference from the behavior of spherical particles. $G$ for the major and minor axes of the spheroid can be analytically derived as

where $a$, $b$, and are the semi-major axis, semi-minor axis, and the aspect ratio ^10^. Assuming independence between the motion in each axis, we can obtain the viscosity as

$$\eta=\frac{\frac{2k_{B}T}{6\pi b}\sum_{i} \frac{1}{G_{i}}}{\sum_{i} MSD_{i}(\tau)}\tau$$

where the degrees of freedom include a major axis and two minor axes.

**References**

1 Barer, R. Determination of dry mass, thickness, solid and water concentration in living cells. *Nature* **172**, 1097-1098 (1953).

2 Popescu, G. *et al.* Optical imaging of cell mass and growth dynamics. *American Journal of Physiology-Cell Physiology* **295**, C538-C544 (2008).

3 Park, C., Shin, S. & Park, Y. Generalized quantification of three-dimensional resolution in optical diffraction tomography using the projection of maximal spatial bandwidths. *JOSA A* **35**, 1891-1898 (2018).

4 Oh, J. *et al.* Three-dimensional label-free observation of individual bacteria upon antibiotic treatment using optical diffraction tomography. *Biomedical optics express* **11**, 1257-1267 (2020).

5 Bechinger, C. *et al.* Active particles in complex and crowded environments. *Reviews of Modern Physics* **88**, 045006 (2016).

6 Portela, R. *et al.* Real-time characterization of the mechanical behaviour of an actively growing bacterial culture by rheology. *arXiv preprint arXiv:1209.5381* (2012).

7 Bartnikowski, M., Wellard, R. M., Woodruff, M. & Klein, T. Tailoring hydrogel viscoelasticity with physical and chemical crosslinking. *Polymers* **7**, 2650-2669 (2015).

8 Perrin, F. Mouvement brownien d'un ellipsoide-I. Dispersion diélectrique pour des molécules ellipsoidales. *J. phys. radium* **5**, 497-511 (1934).

9 Perrin, F. Mouvement Brownien d'un ellipsoide (II). Rotation libre et dépolarisation des fluorescences. Translation et diffusion de molécules ellipsoidales. *Journal de Physique et le Radium* **7**, 1-11 (1936).

10 Happel, J. & Brenner, H. *Low Reynolds number hydrodynamics: with special applications to particulate media*. Vol. 1 (Springer Science & Business Media, 2012).
